# Ovarian Remnant Syndrome in Bitches and Queens: Clinical Aspects and Potential Neoplastic Transformations

**DOI:** 10.3390/ani15213106

**Published:** 2025-10-26

**Authors:** Daniele Zambelli, Giulia Ballotta, Dina Guerra, Marco Cunto

**Affiliations:** Department of Veterinary Medical Sciences, University of Bologna, Ozzano dell’ Emilia, 40126 Bologna, Italymarco.cunto@unibo.it (M.C.)

**Keywords:** ovarian remnant syndrome, dogs, cats, gonadectomy, sterilization complications, reproductive disorders, progesterone assay, vaginal cytology, ultrasonography, veterinary surgery

## Abstract

**Simple Summary:**

Ovarian remnant syndrome (ORS) is a postoperative complication occurring in dogs and cats following gonadectomy, caused by incomplete removal or accidental revascularization of ovarian tissue during sterilization. Affected animals typically present with recurrent signs of oestrus despite being previously spayed. Untreated ORS can lead to serious health issues such as stump pyometra, mammary gland tumours, and ovarian tumours, including granulosa cell tumours. This retrospective study aimed to evaluate signalment, clinical presentation, diagnostic methods, and treatment outcomes in animals diagnosed with ORS referred to the University Veterinary Hospital of Bologna, Italy. A total of 93 cases were reviewed of 70 dogs and 23 cats of various breeds and body size, with a higher incidence observed in dogs. Diagnosis was based on clinical signs and confirmed through vaginal cytology, serum progesterone assay, ultrasonography, and histopathological analysis of excised tissue. Surgical removal of the remnant tissue was the only curative treatment. Optimal outcomes were obtained when revision surgery was performed during hormonally active phases (follicular or luteal), with wide excision of the ovarian pedicle and surrounding scar tissue. These findings emphasize the importance of meticulous surgical technique and early recognition of ORS to improve long-term outcomes and animal welfare.

**Abstract:**

Ovarian remnant syndrome (ORS) is a recognized postoperative complication in spayed dogs and cats, resulting from incomplete excision or inadvertent revascularisation of ovarian tissue during gonadectomy. Affected animals typically exhibit recurrent oestrous behaviour and may develop serious sequelae, including stump pyometra, mammary neoplasia, and granulosa cell tumours. This retrospective study evaluated 93 cases (70 dogs, 23 cats) diagnosed with ORS referred to the University Veterinary Hospital of Bologna, Italy, focusing on signalment, clinical presentation, diagnostic protocols, and treatment outcomes. Diagnosis relied on a multimodal approach combining clinical history, vaginal cytology, serum progesterone assays, ultrasonography, and histopathological examination of excised tissue. Surgical excision of residual ovarian tissue was the only curative treatment, with improved outcomes when performed during hormonally active phases of the oestrous cycle to optimize remnant localisation. Histopathology confirmed ovarian tissue in the majority of cases, with neoplastic transformation identified in 10% of dogs. Bilateral ovarian remnants were more prevalent than previously reported. Surgical revision was complicated by adhesions involving vital abdominal structures, emphasizing the need for meticulous technique. These findings highlight the critical importance of precise surgical technique during initial gonadectomy, early recognition of ORS, and comprehensive surgical management to prevent severe complications and promote companion animal welfare.

## 1. Introduction

Ovarian remnant syndrome (ORS) is defined as the presence of functional ovarian tissue associated with clinical signs of oestrus in a previously spayed bitch or queen [1,2]. First described in 1973 by Pearson [3], ORS is not considered a pathological condition in itself, but rather a complication of gonadectomy resulting either from the incomplete removal of one or both ovaries, or from the revascularization of ovarian tissue accidentally dropped into the peritoneal cavity during surgery, which can revitalize and become functional [1,4,5,6]. Additionally, some authors have reported that the presence of accessory or ectopic ovarian tissue within the ovary or broad ligaments may be another possible cause of ORS [7,8,9,10,11]. Accessory or ectopic ovarian tissue is typically small and separated from the normal ovary by connective tissue. The accessory ovary may become functional when normal ovary is removed [8]. Although this condition has been documented cows, and woman [8], in cats only a single case report exists [12]. However, no histological examination was provided in that case, making it impossible to confirm the true ovarian nature of the tissue. Moreover, it has been documented that in approximately 2% of cats, adrenal nodules can be found within ligament, and these may grossly resemble ovarian tissue [13]. Therefore, due to the lack of histological confirmation and the potential for macroscopic misidentification, there is currently no definitive evidence supporting the existence of true accessory ovarian tissue in cats. Several risk factors have been considered over the years as potential causes of ovarian remnant syndrome (ORS). However, intrinsic characteristics of the subject such as age at the time of spaying, obesity, thoracic conformation, or the surgeon’s experience are not currently considered as risk factors [1,7].

Animals with ORS are typically presented to the veterinarian due to signs or oestrus behaviour. Some bitches may show clinical pseudocyesis in absence of a previously observed oestrus [1,3,7,9]. Complication associated with ORS can include bilateral alopecia and dermal hyperpigmentation, pyometra or stump pyometra, mammary masses, bone marrow aplasia due to prolonged steroid hormone exposure, recurrent urinary tract infection, and ovarian neoplasia, and progesterone-related diabetes mellitus [4,14,15,16,17,18,19]. The presence of remnant ovarian tissue may negatively affect the animal’s quality of life and even reduce lifespan. Moreover, it appears to predispose to the development of ovarian neoplasms. In fact, the incidence of tumours arising from ovarian remnants seems to be higher than that reported in sexually intact female dogs [19]. Among these, sex-cord stromal tumours are the most commonly described although other tumour types have also been reported [17,19,20,21,22,23]. The time between sterilization and the return to oestrus can vary, but one report suggested that the cycles may resemble normal oestrous cycles in length [1,24,25].

A presumptive diagnosis of ORS could be made based on history, clinical signs, vaginal cytology, progesterone levels, and ultrasound examination [1,18,19,26,27]. Vaginal cytology is the most rapid and cost-effective method for presumptive diagnosis in animals showing signs of oestrus, as it permits to detect estrogen activity through the presence of keratinize epithelial cells in a vaginal smear [1]. In dog, progesterone measurement could indicate the presence of corpora lutea; therefore, it can be a useful indicator for functional ovarian tissue, while it is less reliable in cats unless ovulation has been induced [1,28]. In recent years, anti- Müllerian hormone (AMH) has emerged as a valuable serum biomarker for ORS in both dogs and cats [29,30,31,32]. Additionally, it can be use when adrenocortical tumours are suspected, due to their potential to synthesize and secrete sex hormone, which may mimic the clinical signs of ORS. The diagnostic usefulness of abdominal ultrasonography in detecting ovarian remnant syndrome remains a subject of debate, as its accuracy can be influenced by the operator’s experience, the ultrasound equipment used, the size of the remnant tissue, and the stage of the oestrus cycle [33,34]. Nevertheless, van Nimwegen et al. [20] reported that transabdominal ultrasonography successfully identified ovarian remnants in 97% of the dogs in their study, with findings confirmed by histopathological examination. If ultrasound examination is inconclusive computed tomography (CT) may enhance the visualization and identification of ovarian remnants, helping to avoid repeated exploratory surgeries or surgical errors [15].

The definitive treatment of ORS consists of surgical excision of the residual ovarian tissue [26]. Histological examination of all the excised tissue is essential to confirm the diagnosis [18]. Medical management with oestrus-suppressing drugs, such as progestogens, androgens or gonadotropin-releasing hormone (GnRH) agonists has been explored but it should be considered only for poor surgical candidates; in general oestrus suppression is not recommended in patient with an ORS due to lack of proven efficacy when prolonged estrus is due to an ovarian tumour and potential serious consequences in case an underlying (reproductive, mammary or bone marrow) condition is present [9,11,26,27].

The aim of this study was to describe and discuss signalment, clinical signs and findings, diagnostic tools, and treatment of ovarian remnant syndrome in small animals. Furthermore, this study aims to investigate clinical implications and complications associated with the potential neoplastic degeneration of ovarian remnants.

## 2. Materials and Methods

Medical records of dogs and cats presented to the Veterinary University Hospital “Giuseppe Gentile” of Department of Veterinary Medical Sciences (DIMEVET) of the University of Bologna between 2002 and 2025 were retrospectively reviewed. The Animal Welfare Committee of the University of Bologna provided a positive ethical and scientific opinion on the publication of the data, certifying that the study did not involve animal experimentation but rather clinical veterinary practice (prot. 208928/2025).

Cases were identified through a retrospective search of the hospital’s electronic medical record System. Records were included if the clinical presentation and diagnostic work-up were consistent with a presumptive diagnosis of ORS. The search was conducted using relevant keywords and diagnostic terms associated with ORS, and all candidate records were manually reviewed to confirm eligibility. A total of 104 medical records of dogs and cats were identified and reviewed. Of these, 76 concerned dogs and 28 cats. Eleven records (6 dogs and 5 cats) were excluded from the study due to missing data. Consequently, 70 dogs and 23 cats were included, provided that their records contained complete information on signalment, medical history, and diagnosis (vaginal cytology, hormonal assay, and diagnostic imaging). As this was a retrospective study, not all cases underwent the same diagnostic pathway. Abdominal ultrasound was performed in all cases, while vaginal cytology and/or hormonal assays (serum progesterone) were available in a subset of cases, depending on clinical indication and availability of data in the medical records. Signalment data included breed, body weight, and age at the time of presentation. All dogs included in the study were classified by size according to their body weight in small (≤10 kg), medium (>10–<25 kg), large (≥25–<40 kg), and giant (≥40 kg), and morphotype according to their breed-specific physical characteristics. Breeds were classified as dolichomorphic (long-limbed, narrow-chested, and long-headed), mesomorphic (moderately proportioned, muscular, and balanced body structure), or brachymorphic (short-limbed, compact, and broad-headed). Mixed-breed dogs, which could not be reliably assigned to any morphotype category, were recorded separately.

Medical history included age at the time of sterilization, surgical technique used for sterilization, the time elapsed between sterilization procedure and clinical presentation, and clinical signs observed at the time of the visit. When available, data on surgical revision procedures, macroscopic appearance of the ovarian remnant tissue, and histopathological findings were also collected. Follow-up information was obtained through telephone interviews with the owner and/or referring veterinarians.

The diagnosis of suspected ovarian remnant syndrome was based on a combination of clinical signs, vaginal cytology, serum progesterone levels, and ultrasonographic evolution of the reproductive tract. In all cases where surgery revision was performed, the diagnosis of ORS was confirmed by histopathological examination of the reproductive tissue removed during surgery.

### 2.1. Ovarian Remnant Surgical Revision Technique

All animals were routinely anesthetized using a non-standardized protocol, previously approved by the attending anaesthesiologist, and positioned in dorsal recumbency. The ventral abdomen was clipped from the xiphoid process to the pubis, and the skin aseptically prepared for surgery. A ventral midline abdominal incision was made, starting approximately 1–2 cm cranial to the umbilical scar and continuing caudally to the pubis. The abdominal cavity was thoroughly explored to identify any ovarian remnants. Curettage of the ovarian region was performed, with wide excision of all tissue involved in adhesions in order to eliminate any residual ovarian tissue that might not be macroscopically detectable. Additionally, the ovarian ligament and/or scar tissue from previous surgery were identified, isolated, and excised along with approximately 0.5–1 cm of surrounding tissue, as close as possible to the caudal pole of the kidney. The round ligament of the uterus was also excised as much as possible. In all cases where the patient had previously undergone ovariectomy or incomplete hysterectomy, the procedure was completed by performing a total hysterectomy.

Regardless of the presence or absence of macroscopic structures suggestive of ovarian remnants, all excised scar tissue from the ovarian area, as well as the apices of the uterine horn, were submitted for histopathological examination. Samples were analyzed by the Pathological Anatomy Service of the DIMEVET of the University of Bologna to assess the presence of any residual gonadal tissue.

### 2.2. Statistical Analysis

Statistical analyses were performed to identify any correlations or significant differences between the potential presence of an ovarian remnant, the anatomical side of the remnant, and various other variables such as animal size and body conformation, location of the ovarian remnant as determined by ultrasound, macroscopic findings during surgery, and histopathological results. The following specific comparisons were made:

(1) Kruskal–Wallis test was used to assess differences among all the following variables: age at first clinical presentation, age at the time of spaying, body weight at presentation, body size category, macroscopic detection of ovarian remnants during surgery, ultrasonographic detection of ovarian remnants, serum progesterone levels.
(2) Pearson correlation was used to assess the strength of association between continuous variables (age at sterilization, age at presentation at our clinic, body weight at time of presentation, type of surgery, vaginal cytology result, oestrus stage, progesterone, presence of ovarian remnant at ultrasound evaluation, at macroscopical exam and histopathological exam, and presence of tumour).

All statistical analyses were performed using IBM SPSS Statistics version 30.0. A significance level of *p* < 0.05 was used for all statistical tests.

Descriptive statistics, such as mean, median, and range, were calculated for continuous variables (e.g., age at presentation, age at sterilization, weight). Frequency percentages were reported for categorical variables (e.g., dog size, location of the ovarian remnant detected by ultrasonographic examination, macroscopic findings during surgery, histopathological findings, and presence of neoplastic tissue).

## 3. Results

Data from ninety-three animals (70 dogs and 23 cats) met the inclusion criteria and were retrospectively reviewed. All animals presented diagnosis or presumptive diagnosis of ORS. The results of the study are presented separately for dogs and cats.

### 3.1. Dog Population

The female dogs’ population consisted of 70 animals of different age, body weight and breeds. At the time of presentation, the mean age was 6.56 ± 4.20 years (range: 0.66–16.41 years), and the mean body weight was 18.63 ± 10.61 kg (range: 2–50 kg). The mean age at the time of spaying was 3.04 ± 2.55 years (range: 0.5–10.41 years). The distribution of breeds included: mixed-breed (37/70, 52.86%), Labrador retriever (9/70, 12.86%), Beagle (4/70, 5.71%), German Shepherd (3/70, 4.29%), Pug (2/70, 2.86%), English Setter (1/70, 1.43%), Irish Setter (1/70, 1.43%) Dachshund (1/70, 1.43%), French Bulldog (1/70, 1.43%), Dalmatian (1/70, 1.43%), Doberman (1/70, 1.43%), Dogue de Bordeaux (1/70, 1.43%), Flat-coat Retriever (1/70, 1.43%), Maltese (1/70, 1.43%), Little Italian Greyhound (1/70, 1.43%), Rhodesian Ridgeback (1/70, 1.43%), Miniature Schnauzer (1/70, 1.43%), Shih-tzu (1/70, 1.43%), Pomeranian (1/70, 1.43%), and Yorkshire terrier (1/70, 1.43%). Based on body weight, 19 dogs (21.14%) were classified as small (≤10 kg), 24 (34.28%) as medium (>10–<25 kg), 26 (37.14%) as large (≥25–<40 kg), and 1 dog (1.44%) as giant (≥40 kg). Regard the body conformation, 7 (10%) were classified as dolichomorphic (English Setter, Dalmatian, Doberman, Flat-coated Retriever, Irish Setter, Italian Greyhound, and Rhodesian Ridgeback), 20 (28.57%) as mesomorphic (Labrador Retriever, Beagle, German Shepherd, Dogue de Bordeaux, Maltese, Miniature Schnauzer, and Yorkshire Terrier) and 6 (8.57%) as brachymorphic (Pug, Dachshund, French Bulldog, Shih-tzu, and Pomeranian), as summarized in Table 1.

A total of 40 dogs (57.14%) underwent ovariectomy. Of these, 2 were treated using the ovarian bursa opening technique, 1 was spayed before puberty, 1 via laparoscopy, and in 1 case only the right ovary had been removed. Twenty dogs (28.57%) had undergone ovariohysterectomy. Surgical history was unavailable in 10 dogs (14.29%). Two dogs (2/70; 2.86%) received hormonal treatment with progestins (Proligestone, Covinan^®^, Intervet Italia S.r.l., Peschiera Borromeo, Milan) following ovariectomy to suppress oestrus signs. In all 70 dogs, the diagnostic workup supported a presumptive diagnosis of ORS. In 13 cases (22.86%), only diagnostic workup data were available, while in the remaining 54 dogs (77.14%), data from the diagnostic workup, surgery, and histopathological examination were available. Of the 70 dogs, 38 (54.29%) exhibited signs of oestrus within 6–7 months following spaying. In 8 dogs (11.43%), oestrus signs appeared one year after surgery; in 4 dogs (5.71%), after two years; in 3 dogs (4.29%), after three years; in 1 dog (1.43%), after six years; and in 2 dogs (2.86%), after 9–10 years. In 15 cases (21.43%), data regarding the timing of onset of oestrus signs after spaying were not available. Data are summarized in Table 2.

In 7 dogs (10%), ORS was diagnosed incidentally during other clinical evaluations, and no overt signs of oestrus were reported. Specifically, ORS was identified in 2 dogs (2.86%) due to clinical signs associated diabetes mellitus, both dogs were found to be in dioestrus at the time of diagnosis, 3 dogs (4.29%) were presented for vaginal prolapse, and 2 dogs (2.86%) with intrauterine fluid accumulation. Seven dogs (10%) exhibited signs of pseudopregnancy with galactorrhoea, without concurrent behavioural signs of oestrus. The remaining 49 dogs (70.00%) showed behavioural and/or physical signs suggestive of oestrus. Among these, 43 dogs (87.76%) had regular oestrus signs, while 6 dogs (12.24%) showed irregular manifestations. The most frequently observed clinical signs included serosanguineous vulvar discharge (40/49, 81.63%), vulvar edema (29/49, 59.18%), and attraction to male dogs (30/49, 61.22%). A summary of clinical presentations is provided in Table 3.

Vaginal cytology was available in 68 out of 70 dogs (97.14%). Cytological findings were consistent with the follicular phase of the oestrus cycle in 40/70 dogs (57.14%), the luteal phase in 19/70 dogs (27.14%), and the anoestrus phase in 9/70 dogs (12.85%). Serum progesterone concentration was measured in 66 dogs (94.28%), with a mean value of 6.21 ± 8.39 ng/mL (range: 0.20–31.20 ng/mL).

Abdominal ultrasonography was performed in all dogs as part of the diagnostic workup. In 54 out of 70 dogs (77.14%), a structure consistent with an ovarian remnant was identified. Specifically, a right-sided remnant was found in 24 of these 54 dogs (44.44%), a left-sided remnant in 14 dogs (25.92%), a and a bilateral remnant in 16 dogs (29.64%). In 16 dogs (22.86%), no ultrasonographically detectable structure suggestive of ovarian tissue was observed. Among these, the suspicion of ORS was based on complementary diagnostic findings. In 10/16 dogs, the diagnosis was supported by vaginal cytology and serum progesterone concentration: 3 dogs were in the luteal phase, with serum progesterone level between 0.20 ng/mL and 23 ng/mL, and 7 were in follicular phase with progesterone concentration ranging from 0.20 ng/mL to 7 ng/mL. In the remaining 5 out of 16 dogs, all of which were in anoestrus with basal serum progesterone levels the suspicion of ORS was based on the ultrasonographic presence of uterine fluid accumulation. In one additional case (1/16), the diagnostic suspicion arose due to clinical signs of galactorrhoea in absence of other identifiable causes. A summary of ultrasound findings is provided in Table 4.

In all 70 dogs, the diagnostic workup led to a presumptive diagnosis of ORS. However, in 12 out of 70 dogs (17.14%), surgical intervention was not pursued due to various reasons, including lack of owner consent or failure to return for treatment. In the remaining 58 dogs (82.86%), surgical exploration was performed to remove the suspected ovarian remnant.

Histopathological evaluation of the excised tissue (consisting of the previous surgical scar tissue in the ovarian region and uterine horn apices) to confirm the presence of gonadal tissue was available for 54 of these dogs. In the remaining 4 dogs, surgery was performed by the referring veterinarian, and no histological data was available.

Among the 54 dogs with complete surgical and macroscopical data, a right-sided ovarian remnant was suspected in 18 dogs (33.33%), a left-sided remnant in 8 dogs (14.81%), and bilateral remnants in 25 dogs (46.3%). In three cases (5.56%) no grossly visible structure compatible with ovarian remnant was detected. Regarding anatomical localization, the remnant was found at the apex of the uterine horn in 7 dogs (12.96%), within the ovarian bursae in 8 dogs (14.81%), and in the ovarian region, corresponding to the site of the previous surgical scar, in 36 dogs (66.66%).

Histopathological analysis confirmed the presence of ovarian tissue in 50 of 54 samples (92.6%). Specifically, in 7 dogs (12.96%) a left-sided remnant was confirmed, in 21 (38.88%) a right-sided remnant, and in 22 (40.74%) bilateral remnants were diagnosed. In 4 cases (7.41%) no ovarian tissue was identified. All confirmed remnants consisted of histologically normal ovarian tissue, except for 8 cases (16%) in which neoplastic changes were observed. These included four right-sided tumours (1 serous papillary cystadenoma, 1 luteoma, and 2 granulosa cell tumours), two left-sided granulosa cell tumours, and two bilateral tumours (1 papillary cystadenoma of the rete ovarii and 1 granulosa cell tumour). Among the dogs diagnosed with ovarian tumours, three were elderly (older than 13 years); of these, two presented with granulosa cell tumours, and the exact timing of sterilization was unknown. The other three were younger than 2 years at the time of presentation and had been spayed approximately one year earlier (two had granulosa cell tumours and one had an adenoma of the rete ovarii). One additional dog was approximately 5 years old, had been spayed one year previously, and was diagnosed with a granulosa cell tumour on the ovarian remnant.

Data about surgery, macroscopically and histopathological findings are summarised in Table 5, while a summary of the data about dog with histologically confirmed ovarian tumours are reported in Table 6.

The Pearson correlation test revealed a significant positive correlation (r: 0.327, *p* < 0.05) between the age at clinical presentation and the presence of an ovarian tumour. A significant negative correlation (r: −0.021, *p* < 0.05) was observed between the age at the time of spaying and the diagnosis of suspected ovarian remnant syndrome (ORS) performed via ultrasound, indicating that dogs spayed at a younger age are less likely to retain ovarian tissue.

Furthermore, a strong positive correlation (respectively, r: 0.588 and *p* < 0.001, r: 0.372 and *p*: 0.008) was found between the ultrasonographic suspicion of an ovarian remnant and both the macroscopic identification of the remnant during surgery and its histopathological confirmation. Conversely, a significant negative correlation (r: −0.283, *p* < 0.049) was observed between the macroscopic identification of an ovarian remnant and the histopathological diagnosis of an ovarian tumour. No significant correlation was found between the presence or suspicion of an ovarian remnant and any of the other variables analyzed in this study (age at sterilization, body weight, animals’ size and body conformation, location of the ovarian remnant detected by ultrasound examination and macroscopic findings during surgery, and histopathological findings).

The Kruskal–Wallis test (*p* < 0.05) revealed statistically significant differences in the following comparisons:

1. The age of onset of oestrus signs post-spaying and the ultrasonographic detection of suspected ovarian remnants.
2. Body weight and the macroscopic visualization of ovarian remnants during surgery.
3. Serum progesterone levels and histopathological confirmation of ORS.
4. Animal size and histopathological confirmation of ORS.
5. The anatomical distribution of the ovarian remnant and the oestrus cycle phase at the time of diagnosis.

### 3.2. Cat Population

The cat population consisted of 23 animals, of which 20 (87%) were Domestic Short-hair and 3 (13%) were Ragdoll. At the time of presentation, the mean age was 2.49 ± 1.82 years (range: 1–9 years), and the mean body weight was 3.85 ± 0.85 kg (range: 2.5–6 kg). The mean age at the time of spaying was 0.87 ± 0.48 years (range: 0.25–2 years). Twenty cats (86.96%) underwent ovariectomy, one (4.34%) underwent ovariohysterectomy, and in two cases (8.7%), no surgical information was available. Only one cat (1/23) received hormonal treatment after ovariectomy to suppress oestrus signs. The diagnostic workout was consistent with suspected ovarian remnant syndrome in all 23 cats.

All cats exhibited oestrus signs (vocalization), consistent with a normal cycling. Of the 23 cats, 15 (65.2%) showed oestrus signs immediately after spaying surgery. Oestrus signs appeared in one cat (4.3%) one-month post-ovariectomy, in another (4.3%) three months post-surgery, in one cat (4.3%) six months post-surgery, in four cats (17.4%) eight months after surgery, and in one cats (4.3%) two years after sterilization.

Vaginal cytology was consistent with the follicular phase of the oestrus cycle in 20/23 cats (86.95%), with the luteal phase in one cat (4.35%), and with the anoestrus phase in two cats (8.7%). Serum progesterone concentration was assessed in 8 cats, with a mean value of 2.51 ± 5.09 ng/mL. In the remaining 15 cats, cytology alone was considered sufficient to confirm estrogenic activity. Ultrasonographic examination was performed in 21 out of 23 cats as part of the diagnostic workup. An ovarian remnant was identified in 18 out of 21 cats (85.72%), while in 3 cats (14.28%) no structure consistent with ovarian tissue was detected; among these, one was in anoestrus and the other two were in the follicular phase. Of the 18 cats with ultrasonographically detected ovarian tissue, 5 (27.78%) had a right-sided remnant, 7 cats (38.89%) had a left-sided remnant, and 6 (33.33%) had bilateral remnants.

Surgical revision was performed in 14 out of 23 cats (60.86%). In the remaining 9 cats, surgery was not pursued due to various reasons, including lack of owner consent. Histopathological evaluation of the excided tissue (consisting of the previously surgical scar tissue in the ovarian region and the apices of the uterine horns) was available for 12 of the 14 surgically treated cats. In the other two cases, surgery was carried out by the referring veterinarian, and histological data was not available. Among the 12 cats with complete surgical and macroscopical data, 1/12 (8.33%) had a suspected ovarian remnant on the right side, 3/12 (25%) on the left side, and the majority, 8/12 (66.67%), had bilateral remnants. Regarding anatomical localization in 1/12 (8.33%), the remnant was located at the apex of the left uterine horn. Another case (1/12, 8.33%) presented remnants within both right and left ovarian bursae, In the remaining 10 cats (83.34%), the remnants were located at the level of the ovarian region. Histopathological analysis confirmed the presence of ovarian tissue in 10 out of 12 samples (83.34%), consistent with the macroscopical findings. Specifically, in 3/12 (41.67%) identified a left-side ovarian remnant, and in 7/12 (58.33%) a bilateral remnant. In one case (1/12, 8.33%), histopathological data was unavailable, and in another (1/12, 8.33%), no ovarian tissue was identified. All confirmed remnants consisted of normal ovarian tissue, except in one case where the right ovarian remnant was histologically normal, but the left side showed an early-stage adenoma of the rete ovarii. This cat was approximately 2 and a half years old and had been spayed two years previously.

Diagnostic findings in the 12 cats for which complete data on diagnostic workup, surgical exploration, and histopathological examination were available are summarised in Table 7.

The Pearson correlation test revealed a significant positive correlation (r: 0.760, *p* < 0.01) between the ultrasound-based suspicion of an ovarian remnant and the macroscopic identification of an ovarian remnant during surgery. In contrast, a significant negative correlation (r: −0.671, *p* < 0.05) was found between the ultrasound suspicion of an ovarian remnant and the presence of an ovarian tumour. No significant correlation was found between the presence or suspicion of an ovarian remnant and any of the other variables analyzed in this study.

The Kruskal–Wallis test (*p* < 0.05) revealed statistically significant differences between the age of spaying and the macroscopic visualization of ovarian remnants during surgery.

## 4. Discussion

Surgical gonadectomy, whether via ovariectomy (OV) or ovariohysterectomy (OVH), is one of the most commonly performed procedures in dogs and cats. It is primarily used to prevent pregnancy, eliminate oestrus cycles, treat reproductive pathologies, and reduce the risk of reproductive tract neoplasia. Although considered generally safe, postoperative complications can occur. One of the rarest complications is ovarian remnant syndrome, reported in both species [1,4,5,6].

The literature offers contrasting views regarding the relative incidence of ORS in dogs versus cats. Most authors suggest a higher frequency in dogs due to the presence of their unique anatomy featuring an ovarian bursa [19,35]. Reported incidence rates in spayed female dogs varies widely, ranging from 0.1% to 43% [1,3,36,37]. For example, Pearson [3] reported ORS in 12 out of 72 bitches (approximately 17%), Wallace [1] in 22%, and Okkens et al. [36] in 47 of 109 dogs (around 43%). In contrast, Muraro et al. [37] documented an incidence of only 0.1%. In cats, ORS is less frequently reported, and some authors suggest that it may be underdiagnosed due to subtler clinical signs of oestrus [1,7]. More recent studies, however, report rates lower than or comparable to dogs [19,35]. A 2020 study from the University of Cairo found an ORS incidence between 0.3 and 0.6% in cats [38], and Mullikin et al. [33] found no significant species difference, though the limited feline sample size limited the statistical value of the analysis. In the present retrospective study, conducted in a single referral hospital, 104 cases of ORS were identified: 76 dogs and 28 cats. Notably, none of these animals had been originally spayed at our institution, preventing us from calculating true incidence rates due to a lack of data on the total number of procedures performed at referring clinics. Nevertheless, the relatively high number of cases suggests that ORS may be more common than previously assumed. Although we are unable to provide species-specific prevalence rates, our findings support more recent literature indicating a higher predisposition in dogs likely due to anatomical differences [19]. Specifically, dogs have more periovarian adipose tissue, which may obscure visibility during surgery. Additionally, compared to cats, the suspensory ligament of the canine ovary has a greater diameter, is less elastic, and more resistant to traction. Furthermore, the deeper abdominal cavity complicates ovarian exteriorization, increasing the risk of incomplete removal.

Since all cases included in this study were referred and the surgical expertise of the veterinarians who had performed surgery is unknown, it was not possible to assess whether surgical expertise played a role. Therefore, we are unable to confirm or refute previous findings suggesting that surgeon experience is not a risk factor for ORS [1,7]. Similarly, the data available was insufficient to evaluate the influence of surgical procedure (OV versus OVH) or approach (e.g., traditional laparotomy, laparoscopy, left/right flank incision, or ovarian bursa opening technique). This limitation was particularly evident in cats, where 86.96% of the cases followed OV. Although the sample size was too small to draw firm conclusions in dogs, some authors have suggested that OV may allow better access and reduce the risk of retained ovarian tissue [39]; our data neither support nor refute this claim. It is more plausible that the surgical technique itself, rather than the procedure type, plays a greater role in preventing ORS. For example, Demirel et al. [16] noted that the flank approach in cats may hinder access to the contralateral ovary. Similarly, we hypothesize that, in dogs, the technique used to open the ovarian bursa may contribute to incomplete resection. Indeed, the canine ovarian bursa is rich in adipose tissue, which hinders complete visualization of the ovary and makes thorough resection more challenging. Additional prospective studies with detailed surgical records are needed to explore these variables.

According to several authors [1,7,39], the reproductive status at the time of surgery (pyometra, endometritis, or pregnancy), does not appear to increase the risk of ORS. Consistent with this, only 6 of the 70 dogs in our study (8.57%) had undergone emergency sterilization, and none of the affected cats had a history of emergency reproductive surgery.

The data obtained from the present study characterize the typical patient profile, whether canine or feline, affected by ovarian remnant syndrome. Consistent with prior literature [1,2,7,19], ORS affected dogs of various breeds and body conformations. However, the interpretation of body conformation related trends is limited, as 52.86% of the dogs were mixed breeds, making it difficult to assess the influence of body type. Among purebreds, mesomorphic type (28.57%) were most frequently represented. These included breeds that, in the authors’ opinion, may be considered technically challenging for spaying due to their body weight and excessive periovarian adipose tissue (e.g., Labrador Retriever, Dogue de Bordeaux), or due to specific anatomical features such as a short, broad ovarian pedicle or a deeper ovarian localization (e.g., Maltese, German Shepherd). Regarding body size at the time of sterilization while some studies report no association between body size and ORS [1,7], others suggest heavier dogs may be at increased risk [20,36,37]. Because body weight at the time of sterilization was unavailable, we could not verify this hypothesis. However, evaluating breed size (small, medium, large), no significant differences emerged, consistent with findings by Mullikin et al. [33]. Concerning the age at sterilization, our data, in contrast with literature [1], suggested that bitches spayed at a younger age appeared less likely to develop ORS. A plausible explanation is the progressive increase in mesometrial fat with age, complicating ovary visualization and removal. In cats, the available literature is limited. Our findings support prior research indicating no clear association between sterilization age and ORS in cats [7,19,40], though most cats in our study were spayed post-puberty. As in other reports [33], 87% of affected queens were Domestic short-hair, but this likely reflects their higher population and spay rates rather than a true breed predisposition.

Clinical signs of ORS are directly associated with the presence of functional ovarian tissue and the resulting hormonal activity. Affected animals typically exhibit oestrous cycles with normal periodicity [1]. In the present study, signs of oestrus were observed in nearly all animals. Specifically, 7 dogs showed indirect signs of reproductive hormonal activity, while 7 others presented only pseudopregnancy. In the remaining 56 dogs, oestrus signs typically recurred within seven months post-spay in 54.29% of cases, matching prior reports [1,7,19,33]. All cat patients displayed signs of oestrus, often characterized by vocalization, which aligns with reports of earlier symptom onset in felines [1,19].

Diagnosis of suspected ORS relies on clinical signs and complementary tests, including vaginal cytology, hormonal assays, and ultrasonography examination [1,19,20,26,28,33]. Vaginal cytology is a simple, rapid, and cost-effective method for indirectly detecting estrogenic activity in both dogs and cats during the follicular phase [1,19,26]. However, cytology alone may not always be sufficient to determine the specific stage of oestrous cycle. In such cases, hormonal assay, particularly the measurement of serum progesterone, is recommended [1,19,28]. Functional ovarian tissue is the principal source of progesterone in both species, and a serum progesterone concentration above 2 ng/mL confirms the presence of active corpora luteus, supports the diagnosis of an ovarian remnant in spayed animals [1]. In the queen, as induced ovulator, baseline progesterone is less useful unless ovulation has occurred [1]. In our study, serum progesterone was measured in 8 of 23 cats. All but one had baseline levels; the exception showed a concentration of 15 ng/mL, confirming the dioestrus phase previously diagnosed via cytology. In dogs, ORS was suspected in 59 of 70 cases using vaginal cytology (available in 68/70 dogs) and serum progesterone concentration (available in 66/77 dogs), with a mean progesterone concentration of 6.21 ± 8.39 ng/mL. In cats, cytology alone confirmed estrogenic activity in 15 of 23 cases; serum progesterone was assessed in 8, confirming luteal phase in one. Oestradiol was not measured due to poor reliability when interpreted on single samples [1]. Luteinizing hormone (LH) and ant-Mullerian hormone (AMH) were not evaluated due to their high cost, the logistical challenges related to shipping sample to specialized external laboratories, and their limited additional diagnostic value when compared to progesterone, particularly in bitches [1,28,29]. AMH testing can be useful in the diagnosis of ORS and, in some cases, ruling out differential diagnoses such as adrenal cortical tumours, as AMH is secreted exclusively by ovarian tissue. However, false negative results may occur when the remnant is largely composed of luteal tissue, which may produce insufficient AMH to be detected in the general circulation, as described by Burgio et al. [28]. Moreover, while AMH may be particular useful when adrenal tumour secretes only oestrogens, its diagnostic value becomes limited if the tumours secrete only progesterone. In such cases, measuring both progesterone and AMH may not allow a clear distinction between ORS and adrenal neoplasia, since both false-negative AMH result due to luteal tissue and a negative AMH result in presence of a steroid-secreting adrenal tumour could lead to diagnostic uncertainty. Therefore, in cases with oestrus signs and vaginal cytology consistent with the luteal phase, serum progesterone measurement remains a more affordable and reliable diagnostic tool to confirm the diagnosis of ORS. Transabdominal ultrasonography represents an important supporting diagnostic tool when an ovarian tissue remnant is suspected [18,19,26]. In the present study, ovarian remnant-like structures were visualized in 54 of 70 dogs (77.14%) and in 18 of 21 cats (85.72%). In the remaining cases (22.86% of dogs and 14.28% of the cats) diagnosis was based on clinical signs, hormone assays, and supportive ultrasonographic findings (e.g., uterine fluid). The present data are consistent with what reported in literature [19,33], which indicates that species or breed size do not significantly affect ultrasonographic detection.

Surgical exploration was performed in 58 dogs (82.86%) and 14 cats (60.86%). Histopathological confirmation was obtained in 50 dogs and 12 cats (two feline samples were unavailable), confirming ORS. These findings highlight the diagnostic accuracy of combined approaches and underscore that false positives can occur, for instance, when suture granulomas mimic remnants [19]. In four dogs, no ovarian tissue was found histologically despite prior ultrasonographic and hormonal indications. This may be attributed to incomplete surgical removal, incomplete submission of excised tissue, or insufficient histological sectioning by the laboratory, which is particularly relevant when investigating for an ovarian remnant that is not macroscopically evident but suspected on clinical grounds. Nevertheless, ultrasonography correlated with surgical findings in 72.41% of dogs and nearly all cats, with histological confirmation in 64% of canine cases. These results support previous studies demonstrating a strong agreement between the ultrasonographic identification of remnant tissue and intraoperative findings, reinforcing the utility of transabdominal ultrasonography in locating ovarian remnants [19,20,33].

Our data suggest that bilateral ovarian remnants may be more common than previously documented in both canine and feline patients, in disagreement with earlier studies reporting a right-sided predominance of ORS due to the cranial and deeper position of the right ovary [1,36,41]. Reported frequencies of right-sided remnants range from 72% to 87.2% [20,36], while bilateral involvement has been observed in approximately 50% of cases in other studies [7,42]. In our study, histopathological analysis in dogs confirmed bilateral remnants in 40.74% of cases, right-sided in 38.88%, and left-sided in 12.96%. These discrepancies may reflect improvements in surgical techniques, improved intraoperative visualization, or population-specific anatomical differences. In cats, histopathology confirmed bilateral remnants in 58.33% and left-sided in 41.67%; no right-sided remnants were confirmed. Although the feline sample size was limited, these findings again suggest a higher incidence of bilateral involvement compared to the literature, which generally reports a slight predominance of unilateral right-sided remnants [42].

Histological evaluation of all excised tissue during surgical revision is essential, not only to confirm the diagnosis, but also to rule out differential diagnoses. In fact, it has been documented that approximately 2% of cats may present with ectopic adrenal nodules located within the broad ligament, which can grossly resemble ovarian tissue during surgery [13]. Moreover, in spayed females, the close anatomical relationship between the ovaries and adrenal glands must be considered, especially when evaluating suspected ORS cases. Functional adrenal cortical tumours can produce sex hormones such as progesterone or estrogens, leading to clinical signs that closely mimic those of ORS. Therefore, in sterilized animals showing signs of estrus, histopathological analysis is not only important to distinguish residual ovarian tissue from ectopic adrenal tissue, but also crucial to differentiate true ORS from hormonally active adrenal neoplasms. Finally histopathological analysis is important because remnant ovarian tissue may undergo neoplastic transformation. Granulosa cell tumours (GCTs) have been found to be the most common ovarian tumours arising from residual ovarian tissue in both dogs and cats [1,17,20,21,22,23]. In the present study, 66 histological evaluations were performed (54 from dogs and 12 from cats). Ovarian tissue was confirmed in 60 cases (50 dogs and 10 cats), among which 9 cases (1 cat and 8 dogs) showed neoplastic transformation. The cat diagnosed with a neoplasm had a rete ovarii adenoma. Among dogs, the incidence of tumours arising from residual ovarian tissue was 16%, which is higher than the reported incidence of 6.25% in sexually intact female dogs [43], consistent with previous findings [19]. Three dogs presented with benign tumours (two rete ovarii adenomas and one luteoma), while five had malignant GCTs, accounting for 10% of the dogs with histological evaluation. This incidence is higher than previously reported by Van Nimwegen [20], who identified 2 GCTs among 32 cases (~6%). A leading hypothesis for the elevated incidence of granulosa cell tumours in these patients implicates persistent hormonal dysregulation. Following ovariectomy, the loss of negative feedback triggers increased pituitary secretion of LH and FSH. Residual ovarian tissue, when present, is exposed to sustained gonadotropin stimulation, promoting granulosa cells proliferation and predisposing these cells to neoplastic transformation [44].

These findings underscore the importance of surgical management in cases of ovarian remnant syndrome (ORS), which remains the treatment of choice due to the potential complications associated with retained ovarian tissue. Although medical therapy may be applicable, it is not curative and does not prevent the adverse effects related to the presence of residual ovarian tissue. Such complications include uterine stump pyometra, mammary neoplasia, GCTs, and systemic comorbidities like diabetes mellitus [1,21,45], the latter observed in 35.71% of the dogs in this study. In accordance with the existing literature [1,3], the authors suggest that the optimal timing for surgical intervention is during the follicular or luteal phase of the oestrous cycle. During these stages, the presence of follicles or corpora lutea, along with increased vascularization of the ovarian pedicle, facilitates the localisation and removal of residua tissue. Moreover, in queens with a suspected ORS, due to their induced ovulatory nature and the short length of the follicular phase, inducing ovulation with gonadorelin or hCG during oestrus, might be considered in order to extend the window available for surgical planning [10].

Caution is essential during surgical revision. The procedure should not be limited to excising only the macroscopically visible remnants. In cases where no obvious tissue is present, the surgical scar at the level of the ovarian pedicle should be excised with as wide a margin as possible, as suggested in previous studies [1,18,41]. Particular attention should be paid to adhesions resulting from previous surgeries to prevent inadvertent injury to vital structures such as the ureters, pancreas, or intestines. In the present study, the most common observed adhesions in dogs involved the uterine stump, which was consistently adherent to the urinary bladder and occasionally to the omentum. Most omental adhesions were associated with the broad ligaments and uterine apex. Pancreatic adhesions were noted in 4 of the 58 dogs, while adhesions to the right-sided jejunum were found in 3 cases. In one dog, perirenal adipose tissue required excision. In cats, adhesions were primarily omental, with a frequency comparable to that reported by Van Nimwegen et al. [20]. Finally, a comprehensive exploration of the abdominal cavity is critical and should extend beyond more cleansing of the surgical sites. Thorough inspection is necessary to ensure complete identification and removal of any remaining ovarian tissue. When revision surgery is successful, clinical signs often resolve entirely within days to weeks, as previously reported [1]. This observation was confirmed in our study, where 100% of the animals undergoing surgical revision showed complete resolution of oestrus-related clinical signs.

## 5. Conclusions

Ovarian remnant syndrome remains a clinically relevant, though often underdiagnosed, postoperative complication following gonadectomy in dogs and cats. This retrospective study confirms that ORS can occur in animals of various breeds, sizes, and body conformations, with a higher prevalence observed in dogs, likely attributable to species-specific anatomical factors. The condition is frequently associated with recurrence of oestrous signs and, in some cases, with significant comorbidities such as pyometra, mammary tumours, and granulosa cell tumours (GCTs). Moreover, in the authors’ opinion, the presence of multiple oestrous cycles, albeit with altered duration and interoestrous intervals, may suggest the persistence of normal, functional residual ovarian tissue. In contrast, continuous or prolonged oestrous signs could indicate the presence of pathological gonadal remnants, such as hormonally active ovarian cysts or neoplasms, or exposure to exogenous hormones. Accurate diagnosis requires a multimodal approach that integrates clinical history, vaginal cytology, hormonal assays, particularly serum progesterone, and transabdominal ultrasonography. Histopathological evaluation remains the diagnostic gold standard, not only for confirmation but also for identifying potential neoplastic transformation. The absence of ovarian tissue in some histological samples, despite positive clinical and diagnostic findings, underscores the necessity of submitting all excised tissue for histopathological examination to confirm the diagnosis and assess the success of surgical treatment. Moreover, it is advisable to request that the pathologist examine multiple histological sections of the submitted samples to enhance the likelihood of detecting even minimal ovarian remnants. Surgical excision remains the only curative option. To enhance surgical outcomes and reduce the likelihood of recurrence, revision surgery should ideally be performed during hormonally active phases of the oestrous cycle, either follicular or luteal, when residual ovarian tissue is more easily identifiable. Wide excision of both ovarian pedicle, including any associated scar tissue, is strongly recommended, even in the absence of grossly visible remnants. Given the frequent presence of adhesions, often involving critical structures such as the ureters, intestines, or pancreas, careful dissection and advanced surgical expertise are essential during revision procedures. Where feasible to ensure complete removal of any potential ovarian remnants, all adhesion, such as those involving the omentum, should also be resected with wide margins. This study emphasizes the importance of meticulous surgical technique during initial gonadectomy, as well as the need for increased clinical awareness to facilitate early recognition and appropriate management of ORS. Future prospective studies with standardized surgical protocols, consistent data collection, and long-term follow-up are necessary to further elucidate risk factors, improve prevention strategies, and optimize both diagnostic and surgical management of ORS in canine and feline patients.

## Figures and Tables

**Table 1:** Distribution of the study population according to morphotype classification.

Morphotype	Included Breeds	Number of Dogs (*n*)	Frequency (%)
Dolichomorphic	English Setter, Dalmatian, Doberman, Flat-coated Retriever, Irish Setter, Italian Greyhound, Rhodesian Ridgeback	7	10.00
Mesomorphic	Labrador Retriever, Beagle, German Shepherd, Dogue de Bordeaux, Maltese, Miniature Schnauzer, Yorkshire Terrier	20	28.57
Brachymorphic	Pug, Dachshund, French Bulldog, Shih-tzu, Pomeranian	6	8.57
Mixed-breed dogs	Not classifiable by standard morphotype	37	52.86
Total	—	70	100.00

**Table 2:** Onset of oestrus signs in 70 dogs with diagnoses of ORS.

Time from Spaying to Onset of Oestrus Signs	Number of Dogs (*n*)	Percentage (%)
≤6–7 months	38	54.29%
1 years	8	11.43%
2 years	4	5.71%
3 years	3	4.29%
6 years	1	1.43%
9–10 years	2	2.86%
Data not available	15	21.43%
TOTAL	70	100%

**Table 3:** Clinical presentation of 70 dogs with diagnoses of Ovarian remnant syndrome.

Clinical Finding	Number of Dogs (*n*)	Percentage (%)	Notes
**Incidental diagnosis** **(no oestrus signs)**	7	10	ORS found during unrelated evaluations
Dioestrus-associated diabetes mellitus	2	2.86	Clinical signs of diabetes
Vaginal prolapse	3	4.29	
Uterine fluid accumulation	2	2.86	
**Pseudopregnancy**	7	10	
**Dog with oestrus signs**	49	70	Included both regular and irregular signs
Regular oestrus signs	43	61.43 (of total)	87.76% of dogs with oestrus signs
Irregular oestrus signs	6	8.57 (of total)	12.24% of dogs with oestrus signs
Serosanguineous vulvar discharge	9 (of dogs with oestrus signs)	81.63 (of dogs with oestrus signs)	
Vulvar edema	29 (of dogs with oestrus signs)	59.18 (of dogs with oestrus signs)	
Attraction to male dogs	30 (of dogs with oestrus signs)	61.22 (of dogs with oestrus signs)	
TOTAL	70	100%	

**Table 4:** Summary of ultrasound findings and complementary diagnostic data in 70 dogs with presumptive ORS.

Finding Category	Number of Dogs (*n*)	Percentage (%)
**Ultrasound findings**		
**Ovarian remnant detected (total)**	54	77.14
Right-sided	24	44.44 *
Left-sided	14	25.92 *
Bilateral	16	29.64 *
**No remnant identified**	16	22.86
**Complementary diagnostics among dogs without ultrasound evidence of ORS (n = 16)**		
**Diagnosis supported by cytology + progesterone:**	10	62.5 **
Luteal phase	3	-
Follicular phase	7	-
**Anoestrus phase (diagnosis based on intrauterine fluid)**	5	32.25 **
**Diagnosis based on galactorrhoea (no other causes)**	1	6.25 **

* Percentages marked based on n = 54 (dogs with ultrasound-detected remnants). ** Percentages marked based on n = 16 (dogs without ultrasound evidence).

**Table 5:** Summary of surgical, macroscopical, and histological findings in 70 dogs with presumptive ORS.

Parameter	Number of Dogs (*n*)	Percentage (%)
**Diagnostic outcome**		
Presumptive diagnosis of ORS (all dogs)	70	100
Dogs not undergoing surgery	12	17.14
Dogs undergoing surgical revision	58	82.86
**Histopathological evaluation available**	54	77.14 (of 70)
**Histopathological evaluation not available**	4	5.71 (of 70)
**Macroscopical remnant localization (n = 54)**		
Right-sided remnant suspected	18	33.33
Left-sided remnant suspected	8	14.81
Bilateral remnant suspected	25	46.30
No grossly visible remnant	3	5.56
**Anatomical localization of remnant (n = 54)**		
Apex of uterine horn	7	12.96
Within ovarian bursae	8	14.81
Ovarian region/surgical scar site	36	66.66
**Histopathological confirmation of ovarian tissue (n = 54)**		
Ovarian tissue confirmed	50	92.60
No ovarian tissue identified	4	7.41
**Site of confirmed remnant (n = 50)**		
Left-sided remnant	7	12.96
Right-sided remnant	21	38.88
Bilateral remnant	22	40.74

**Table 6:** Summary of dogs with histologically confirmed ovarian tumours. “Unknown” indicates that the age at spaying was not available in the medical records. Laterality refers to the side(s) where the tumour was identified.

Case	Age at Diagnosis (Years)	Age at Spaying (Years)	Animal Size	Tumour Type	Laterality
**1**	15	Unknown	Small	Granulosa cell tumour	Bilateral
**2**	16	Unknown	Medium	Granulosa cell tumour	Left
**3**	13	Unknown	Large	Luteoma	Right
**4**	2	1	Large	Granulosa cell tumour	Right
**5**	1.91	1	Medium	Granulosa cell tumour	Right
**6**	1.58	1	Medium	Adenoma of the rete ovarii	Bilateral
**7**	5	4	Medium	Granulosa cell tumour	Left
**8**	Not specified	Not specified	Large	Serous papillary cystadenoma	Right

**Table 7:** Diagnostic findings in the 12 cats for which complete data on diagnostic workup, surgical exploration, and histopathological examination were available.

Case	Breed	Type of Surgery	Vaginal Cytology	P4 (ng/mL)	Ultrasound Findings	Macroscopical Localization	Histological Findings
**1**	Domestic Shorthair	OV	Follicular Phase	No	The apex of the left uterine horn	The apex of the left uterine horn	Normal gonadic tissue
**2**	Domestic Shorthair	OV	Follicular Phase	1.8	The apex of the right uterine horn	Right and left ovarian bursae	Right side: normal ovarian tissue; left side adenoma of the rete ovarii
**3**	Domestic Shorthair	OV	Follicular Phase	No	No ovarian tissue detected	Right ovarian ligament ovarian remnant	Missed
**4**	Domestic Shorthair	OV	Follicular Phase	No	Bilateral ovarian	Bilateral ovarian ligament presence of ovarian remnant	Bilateral ovarian tissue
**5**	Domestic Shorthair	OV	Luteal phase	15	Left-side ovary	Left ovarian ligament	Left normal ovarian tissue
**6**	Domestic Shorthair	OV	Follicular Phase	1.87	Left-side ovary	Left ovarian ligament	Left normal ovarian tissue
**7**	Domestic Shorthair	OV	Follicular Phase	No	Left-side ovary	Left ovarian ligament	Left side normal ovarian tissue
**8**	Domestic Shorthair	OV	Follicular Phase	No	Bilateral ovarian tissue	Bilateral ovarian ligament	Bilateral normal ovarian tissue
**9**	Domestic Shorthair	OV	Follicular Phase	No	Bilateral ovarian tissue	Bilateral ovarian ligament	Bilateral normal ovarian tissue
**10**	Domestic Shorthair	OV	Follicular Phase	No	Bilateral Ovarian tissue	Bilateral ovarian tissue	No ovarian tissue detects
**11**	Domestic Shorthair	OV	Follicular Phase	0.53	Bilateral Ovarian tissue	Bilateral ovarian tissue ligament	Bilateral ovarian tissue
**12**	Domestic Shorthair	OV	Follicular Phase	No	Left side ovarian tissue	Bilateral ovarian tissue	Bilateral ovarian tissue

## Data Availability

The original contributions presented in this study are included in the article. Further inquiries can be directed to the corresponding author.

## References

[1] Wallace M.S. (1991). The ovarian remnant syndrome in the bitch and queen. Vet. Clin. N. Am. Small Anim. Pract..

[2] Muram D., Drouin P. (1982). Ovarian remnant syndrome. Med. Assoc. J..

[3] Pearson H. (1973). The complications of ovariohysterectomy in the bitch. J. Small Anim. Pract..

[4] DeNardo G.A., Becker K., Brown N.O., Dobbins S. (2001). Ovarian remnant syndrome: Revascularization of free-floating ovarian tissue in the feline abdominal cavity. J. Am. Anim. Hosp. Assoc..

[5] Fontes G.S., McCarthy R.J. (2020). Ovarian remnant syndrome in a cat with ovarian tissue in the omentum. J. Am. Vet. Med. Assoc..

[6] Diel de Amorim M., Lerer A., Durzi T., Foster R.A., Gartley C.J. (2018). Identification of ectopic ovotestis in a dog with XX ovotesticular, SRY-negative, disorder of sexual development. Reprod. Domest. Anim. Zuchthyg..

[7] Miller D.M. (1995). Ovarian remnant syndrome in dogs and cats: 46 cases (1988–1992). J. Vet. Diagn. Invest..

[8] McEntee K., McEntee K. (1990). Congenital anomalies. Reproductive Pathology of Domestic Mammals.

[9] Johnston S.D., Kustritz M.V., Olson P.N.S., Kersey R. (2001). Disorders of the Canine Ovary. Canine and Feline Threriogenology.

[10] Johnston S.D., Kustritz M.V.R., Olson P.N.S. (2001). Disordes of the Feline Ovaries. Canine and Feline Theriogenology.

[11] Feldman E.C., Nelson R.W., Feldman E.C., Nelson R.W. (2004). Ovarian remnant syndrome. Canine and Feline Endocrinology and Reproduction.

[12] (1977). Third ovary in a cat. Mod. Vet. Pract..

[13] Altera K.P., Miller L.N. (1986). Recognition of feline parovarian nodules as ectopic adrenocortical tissue. J. Am. Vet. Med. Assoc..

[14] Pöppl A.G., Mottin T.S., González F.H. (2013). Diabetes mellitus remission after resolution of inflammatory and progesterone-related conditions in bitches. Res. Vet. Sci..

[15] Ganz S., Wehrend A. (2021). Uptake of exogenous estrogen as a differential diagnosis of ovarian-remnant-syndrome in a bitch: A case report. BMC Vet. Res..

[16] Demirel M.A., Acar D.B. (2012). Ovarian remnant syndrome and uterine stump pyometra in three queens. J. Feline Med. Surg..

[17] Pluhar G.E., Memon M.A., Wheaton L.G. (1995). Granulosa cell tumor in an ovariohysterectomized dog. J. Am. Vet. Med. Assoc..

[18] Sangster C. (2005). Ovarian remnant syndrome in a 5-year-old bitch. Can. Vet. J..

[19] Ball R.L., Birchard S.J., May L.R., Threlfall W.R., Young G.S. (2010). Ovarian remnant syndrome in dogs and cats: 21 cases (2000–2007). J. Am. Vet. Med. Assoc..

[20] van Nimwegen S.A., Van Goethem B., de Gier J., Kirpensteijn J. (2018). A laparoscopic approach for removal of ovarian remnant tissue in 32 dogs. BMC Vet. Res..

[21] Sivacolundhu R.K., O’Hara A.J., Read R.A. (2001). Granulosa cell tumour in two speyed bitches. Aust. Vet. J..

[22] Phipps W.E., Goodman A.R., Sullivan M. (2016). Ovarian remnant removal using minimally invasive laparoscopic techniques in four dogs. J. Small Anim. Pract..

[23] Rota A., Tursi M., Zabarino S., Appino S. (2013). Monophasic teratoma of the ovarian remnant in a bitch. Reprod. Domest. Anim. Zuchthyg..

[24] Concannon P.W., Lein D.H., Kirk R.W. (1989). Hormonal and clinical correlates of ovarian cycles, ovulation, pseudopregnancy, and pregnancy in dogs. Current Veterinary Therapy.

[25] Feldman E.C., Nelson R.W. (1987). Canine and Feline Endocrinology and Reproduction.

[26] Sontas B., Gurbulak K., Ekici H. (2007). Ovarian remnant syndrome in the bitch: A literature review. Arch. Med. Vet..

[27] Romagnoli S. Ovarian remnant syndrome. Proceedings of the Fourth EVSSAR Congress.

[28] Burgio M., Ferré-Dolcet L., Carbonari A., Frattina L., Rizzo A., Cicirelli V. (2025). Unexpected Basal Anti-Müllerian Hormone Concentrations in a 6-Year-Old Bitch Presenting an Ovarian Remnant. Animals.

[29] Place N.J., Hansen B.S., Cheraskin J.L., Cudney S.E., Flanders J.A., Newmark A.D., Barry B., Scarlett J.M. (2011). Measurement of serum anti-Müllerian hormone concentration in female dogs and cats before and after ovariohysterectomy. J. Vet. Diagn. Invest..

[30] Place N.J., Cheraskin J.L., Hansen B.S. (2019). Evaluation of combined assessments of serum anti-Müllerian hormone and progesterone concentrations for the diagnosis of ovarian remnant syndrome in dogs. J. Am. Vet. Med. Assoc..

[31] Turna Yilmaz Ö., Toydemir T.S., Kirsan I., Gunay Ucmak Z., Caliskan Karacam E. (2015). Anti-Müllerian hormone as a diagnostic tool for ovarian remnant syndrome in bitches. Vet. Res. Commun..

[32] Karakas Alkan K., Ceylan A., Alkan H., Ozen D., Bayraktaroglu A.G., Kaymaz M. (2019). Immunohistochemical and qPCR determination of the expression and serum level of anti-Müllerian hormone in pre-pubertal, intact and ovarian remnant syndrome detected bitches. Reprod. Domest. Anim. Zuchthyg..

[33] Mullikin K., Byron M., Chen J., Cheong S.H., Gartley C., Diel de Amorim M. (2022). Ovarian remnant syndrome in small animals: Case series. Clin. Theriogenology.

[34] Devender K., Akshay K., Pratyush K., Chhote L.Y., Yadav S.P. (2018). Ovarian remnant syndrome: A review. J. Entomol. Zool. Stud..

[35] Naiman J.H., Mayhew P.D., Steffey M.A., Culp W.T., Runge J.J., Singh A. (2014). Laparoscopic treatment of ovarian remnant syndrome in dogs and cats: 7 cases (2010–2013). J. Am. Vet. Med. Assoc..

[36] Okkens A.C., Dieleman S.J., v d Gaag I. (1981). [Gynaecological complications following ovariohysterectomy in dogs, due to: (1) Partial removal of the ovaries. (2) Inflammation of the uterocervical stump (author’s transl)]. Tijdschr. Voor Diergeneeskd..

[37] Muraro L., White R.S. (2014). Complications of ovariohysterectomy procedures performed in 1880 dogs. Tierarztl Prax Ausg K Kleintiere Heimtiere.

[38] Farghali H.A.M., Senna N.A., Khattab M.S., Shalaby R.K.I. (2020). Prevalence of Most Common Feline Genital Surgical Affections in Teaching Veterinary Hospital, Cairo University, Egypt and Different Pet Clinics. Adv. Anim. Vet. Sci..

[39] van Goethem B., Schaefers-Okkens A., Kirpensteijn J. (2006). Making a rational choice between ovariectomy and ovariohysterectomy in the dog: A discussion of the benefits of either technique. Vet. Surg..

[40] Little S. (2011). Feline reproduction: Problems and clinical challenges. J. Feline Med. Surg..

[41] Stone E.A. (2003). Textbook of Small Animal Surgery.

[42] Heffelfinger D.J. (2006). Ovarian remnant in a 2-year-old queen. Can. Vet. J..

[43] Dow C. (1960). Ovarian abnormalities in the bitch. J. Comp. Pathol..

[44] Buijtels J.J., de Gier J., Kooistra H.S., Kroeze E.J., Okkens A.C. (2010). Alterations of the pituitary-ovarian axis in dogs with a functional granulosa cell tumor. Theriogenology.

[45] Moosavian H., Fazli M. (2022). Hypercholesterolemia and insulin resistance associated with ovarian remnant syndrome in a diabetic dog: Case report. Vet. Res. Forum.

